# Bilateral versus unilateral balloon pulmonary angioplasty for inoperable chronic thromboembolic pulmonary hypertension

**DOI:** 10.1186/s12931-022-02017-6

**Published:** 2022-05-07

**Authors:** Cheng Hong, Jianmin Lu, Xiaofeng Wu, Wenliang Guo, Jielong Lin, Riken Chen, Haimin Liu, Haiming Chen, Yongxia Lei, Jian Wang, Yue Zhong, Chunying Zhuang, Xinlu Wang

**Affiliations:** 1 State Key Laboratory of Respiratory Diseases, National Clinical Research Center for Respiratory Disease, Guangzhou Institute of Respiratory Health, The First Affiliated Hospital of Guangzhou Medical University, Guangzhou, Guangdong China; 2 Department of Radiology, The First Affiliated Hospital of Guangzhou Medical University, Guangzhou, Guangdong China; 3 Division of Translational and Regenerative Medicine, The University of Arizona College of Medicine, Tucson, AZ USA; 4 Guangzhou Medical University, Guangzhou, Guangdong China; 5 Department of Nuclear Medicine, The First Affiliated Hospital of Guangzhou Medical University, 151 Yanjiang Road, Guangzhou, 510010 Guangdong Province China

**Keywords:** Chronic thromboembolic pulmonary hypertension, Balloon pulmonary angioplasty, Complication, Treatment

## Abstract

**Background:**

To evaluate the safety and efficacy of bilateral balloon pulmonary angioplasty (BPA) as compared with unilateral BPA for patients with inoperable chronic thromboembolic pulmonary hypertension (CTEPH).

**Method:**

We reviewed 210 consecutive BPA sessions for 92 CTEPH patients, including 124 unilateral BPA sessions and 86 bilateral BPA sessions. Radiation exposure, operation details, lesions characteristics and the occurrence of complications were compared between unilateral BPA and bilateral BPA. 131 BPA sessions with a hemodynamics follow-up were included for efficacy analysis, in which hemodynamics changes were compared. Logistic regression analysis was used to identify factors associated with the occurrence of complications.

**Result:**

Bilateral BPA treated more lobes, arteries and lesions [3 (2, 4) vs. 2 (1, 3) lobes, p < 0.001; 8 (5.5, 10) vs. 6 (4, 8) vessels, p = 0.003; 9 (7, 12) vs. 8 (5, 10) lesions, p = 0.01] in one single session than unilateral BPA in a comparable operation duration and amount of contrast media given. Overall, the occurrence of complications was similar between bilateral BPA and unilateral BPA [9 (10.5%) vs. 12 (9.7%), p = 0.83]. Hemodynamics effects didn’t differ significantly between bilateral BPA and unilateral BPA in a single session [mPAP, − 4.5 ± 8.6 vs. − 3.6 ± 7.3 mmHg, p = 0.52; PVR, − 1.1 (− 3.5, 0.8) vs. − 1.8 (− 5.2, 0.3) Wood units, p = 0.21]. For the initial BPA session, bilateral BPA also treated more lobes, arteries and lesions than unilateral BPA [3 (2, 4) vs. 2 (1, 2) lobes, p < 0.001; 8.0 (5.8, 9.3) vs. 6.0 (4.0, 8.0) vessels, p = 0.04; 9 (6, 12) vs. 7 (4, 10) lesions, p = 0.02]. The occurrence of complications was also similar [5 (13.2%) vs. 5 (9.3%), p = 0.80], even in patients with poor baseline hemodynamics. Univariate regression analysis reveals the number of lobes treated/session, but not bilateral BPA, as predictive factors of complications.

**Conclusion:**

Bilateral BPA may be safely and effectively performed in patients with CTEPH without increasing operation duration and radiation burden, even in patients with unfavorable baseline hemodynamics.

## Background

Chronic thromboembolic pulmonary hypertension (CTEPH), classified as World Health Organization (WHO) group IV pulmonary hypertension, is a progressive pulmonary vascular disease characterized by pulmonary artery stenosis or obstructions resulting from incompletely resolved thromboembolic material [1]. Elevated pulmonary vascular resistance (PVR), along with severe pulmonary hypertension might eventually lead to right heart failure and death if left untreated [2]. To date, pulmonary endarterectomy (PEA) remains the treatment of choice for most patients with CTEPH [3]. However, due to the distal thromboembolism inaccessible to endarterectomy or patients’ comorbidities, only about 60% of patients are eligible for PEA [4]. While pulmonary arterial hypertension (PAH) targeted therapy has been proved to improve hemodynamics and exercise capacity, stenosis or obstructions lead by unresolved thromboembolism remained untreated [5, 6].

In the aim of dilating narrowed or obstructed segmental and sub-segmental pulmonary arteries, balloon pulmonary angioplasty (BPA) has emerged as a novel endovascular treatment for patients with inoperable CTEPH, significantly improving patients’ hemodynamics, exercise capacity and right heart function, with a relatively low rate of complications [7]. Current BPA practice involves the evaluation of the feasibility of BPA by an expert CTEPH team; the identification of pulmonary artery lesions suitable for BPA; and the dilatation of target lesions with appropriate balloons to restore pulmonary blood flow. In order to achieve favorable hemodynamic and clinical outcomes, staged procedures with repeated interventions and dilatations are needed [7].

At present, the process of a BPA session is determined mainly by operators’ experience, patient condition, the amount of contrast media given, time of procedure, and the number and type of treated lesions [8]. Generally, segmental and sub-segmental pulmonary arteries of one single lobe (preferably right lower lobe in the initial session) are treated in a single BPA session in order to curb the risk of perioperative complications. While more than one lobe may be treated in one BPA session in patients with relatively low mean pulmonary artery pressure (mPAP), most cohort studies didn’t state clearly the criterion of limiting the number of lobes treated for one BPA session [9–11]. Is it more “dangerous” to treat two or even more lobes in one single BPA session? Is it feasible to treat both lungs in one single BPA session? These questions remained unanswered.

In the present study, we sought to comprehensively assess the safety and efficacy of bilateral BPA as compared with unilateral BPA for patients with inoperable CTEPH in a single center experience.

## Methods

This retrospective study was approved by the institutional review board of the First Affiliated Hospital of Guangzhou Medical University (No. 202172). Written informed consent was obtained from all patients in this study.

### Patients selection

From January 2019 to November 2021, 92 patients with confirmed CTEPH who underwent at least one BPA session in our center were included. Patients underwent a complete workup, including ventilation/perfusion lung scan, computed tomographic pulmonary angiography, right heart catheterization, as well as medical history and comorbidity assessment. For patients with suspicious CTEPH, the diagnosis of CTEPH and the judgement of operability were evaluated by a multidisciplinary CTEPH team including interventional radiologists, surgeons for PEA, radiologists experienced in pulmonary vascular imaging and pulmonologists with expertise in PH, as recommended by the current guidelines [3].

### Balloon pulmonary angioplasty

We performed BPA as a staged procedure in a standard fashion as described previously [12]. Briefly, BPA was performed via right femoral venous access. A 70-cm 7-French vascular sheath was inserted into the vein, through which a 6-French guiding catheter was advanced into the target pulmonary artery. We selected target vessels appropriate for angioplasty and target lesions with webs, filling defects or complete occlusion based on selective pulmonary angiography and optical coherence tomography [13]. A 0.014-in. guidewire (SION Blue, ASAHI) was passed through the target lesion, and the target lesion was dilated to an appropriate size by multiple balloon inflations manually using semi-compliant balloons (diameter range 2.0–6.0 mm) depending on vessel diameter. During BPA procedures, oxygen was given at a flow rate of 3–4 L/min in all of the patients. The operation duration, the amount of contrast media given per session, the dose area product (DAP) per session, the number of lesions, arteries, lobes treated per session and the occurrence of complications were documented for following analysis. In the present study, lesion types were classified into the following: ring-like stenosis lesion, web lesion, subtotal occlusion lesion, total occlusion lesion, and tortuous lesion, according to previous study [14].

We didn’t limit the number of lobes treated per session. Arteries of both lungs would be treated in one BPA session if necessary. Ring-like stenosis and web lesions were targeted in priority. Generally, 2–12 segmental or sub-segmental arteries were treated in each session according to patient condition and the amount of contrast media given (< 300 ml). Two BPA sessions were performed at 2–3 months intervals until mPAP below 30 mmHg was achieved, and/or when all accessible lesions have been treated.

### Patient evaluation and BPA related complications

Upon admission, WHO functional class and serum level of N-terminal pro-B-type natriuretic peptide (NT-proBNP) were evaluated in all patients. Right heart catheterization (RHC) was performed using a Swan-Ganz catheter before and immediately after each BPA procedure, and at follow-up. Hemodynamic parameters including pulmonary artery pressure (PAP), pulmonary arterial wedge pressure, mean right atrial pressure, pulmonary arterial oxygen saturation (PASO_2_) and mixed venous oxygen saturation (MVSO_2_) were measured. cardiac output (CO) was assessed by the thermodilution method and cardiac index (CI) and PVR were calculated based on previous measurements. Efficacy of each BPA session was determined by changes of hemodynamic parameters and NT-proBNP before and after a single BPA session, evaluated before the present BPA session and the next BPA session, or at follow-up.

In patients who completed the BPA treatments, we examined hemodynamics parameters, NT-proBNP and exercise capacity and compared the data before the first BPA session and those at follow-up after the last BPA session, giving an overall view of the efficacy of BPA treatments.

In the present study, complications related to BPA were defined in the reference of the definition and classification of complications related to BPA procedures proposed during the 6th World Symposium on Pulmonary Hypertension [1]. During the procedure: (1) Hemoptysis; (2) pulmonary artery perforation (presence of extravasation of contrast, hypoxaemia, cough, tachycardia, increased pulmonary arterial pressure); (3) pulmonary artery dissection; (4) Allergic reaction to contrast; (5) Adverse reaction to conscious sedation/local anaesthesia. After the procedure: (6) lung injury (presence of lung opacities on chest X-ray and/or CT scan with or without hypoxaemia and with or without haemoptysis; (7) the use of mechanical ventilation or extracorporeal membrane oxygenation; (8) renal dysfunction. The occurrence of complications was compared between unilateral BPA and bilateral BPA.

### Statistical analysis

Continuous variables are expressed as mean ± standard deviation or median and interquartile range according to variable distribution. Categorical variables, such as gender, WHO functional class and use of PAH targeted therapy or incidence of complications were expressed as number and percentage and were compared using the χ^2^ test for independence or Fisher’s exact test. Differences in continuous variables, such as hemodynamic parameters, were compared using the independent Student’s t-test for normally distributed variables and the Mann–Whitney U test for non-normally distributed variables. Logistic regression analysis was used to examine the effect of each predictive variable on the occurrence of complications. Variables with a p-value < 0.01 were included in the multivariate analysis. All statistical analyses were performed using SPSS statistics 25.0 (IBM, Armonk, NY, USA). A p-value < 0.05 was considered statistically significant.

## Results

### Patient characteristics and BPA procedures

**Fig. 1:**
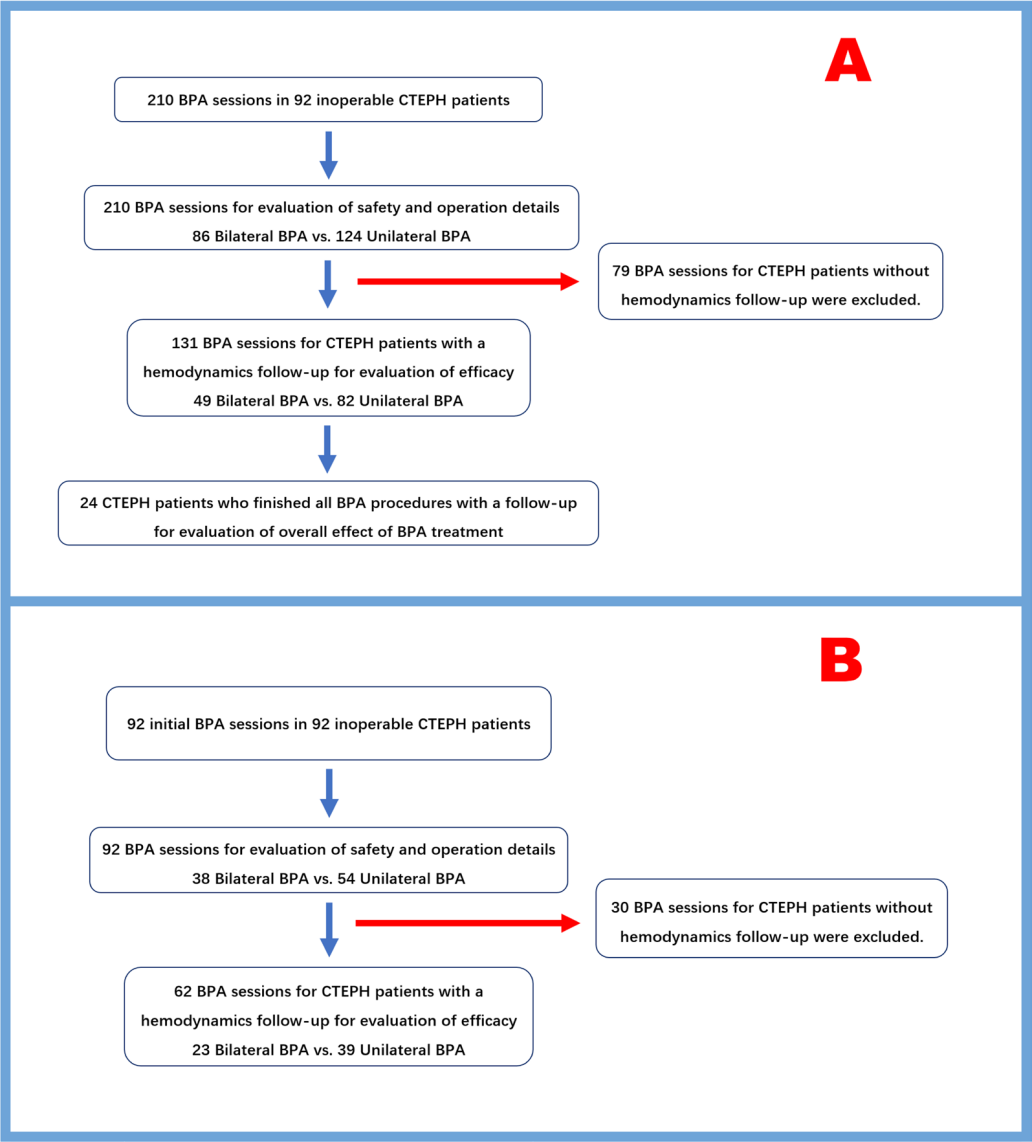
Study flow chart. *BPA* balloon pulmonary angioplasty, *CTEPH* chronic thromboembolic pulmonary hypertension

From January 2019 to November 2021, 92 patients with inoperable CTEPH who underwent at least one BPA session in our center were included. In total, we analyzed data from 210 BPA sessions for evaluation of the efficacy or safety of bilateral BPA as compared with unilateral BPA (Fig. 1A). The patients baseline characteristics before the first BPA are shown in Table 1. In 92 CTEPH patients, mean age was 59 ± 12 year-old, and 63 of them (68%) were female. A total of 210 BPA sessions were performed [2 (1,3) sessions/patient], and the interval between each BPA session was 80 (48,131) days. Most patients had a WHO functional class of II (45, 48.9%) or III (36, 39.1%) at the time of the first BPA session. 50 (54.3%) patients have received PAH targeted therapy before their first BPA session, and all patients received anticoagulant therapy with warfarin or new oral anticoagulants including Rivaroxaban or Dabigatran.

**Table 1:** Patient characteristics before BPA

	CTEPH (N = 92)
Age (years)	59 ± 12
Female (%)	63 (68%)
Number of BPA sessions/patient	2 (1, 3)
Total BPA sessions	210
Interval between BPA (days)	80 (48, 131)
Hemodynamics	
Heart rate	81 ± 13
Mean RAP (mmHg)	7.4 ± 5.0
Systolic PAP (mmHg)	75.5 ± 25.6
Diastolic PAP (mmHg)	24.8 ± 9.0
Mean PAP (mmHg)	43.0 ± 13.6
PVR (Wood Unit)	10.3 ± 6.7
Cardiac output (L/min)	4.1 ± 1.8
Cardiac index (L/min/m^2^)	2.6 ± 1.1
PASO_2_ (%)	62.5 ± 10.2
MVSO_2_ (%)	93.9 ± 4.4
NT-proBNP (pg/ml)	541.1 (120.0, 1823.8)
Exercise capacity	
WHO functional class (I/II/III/IV, %)	7/45/36/4 (7.6/48.9/39.1/4.3)
PAH targeted therapy (%)	50 (54.3%)
Riociguat	28 (30.4)
Macitentan	11 (12.0)
Treprostinil	10 (10.9)
Tadalafil	8 (8.7)
Sildenafil	7 (7.6)
Ambrisentan	5 (5.4)
Bosentan	4 (4.3)
Anticoagulation (%)	92 (100%)
Warfarin	19 (20.7)
Rivaroxaban	64 (69.6)
Dabigatran	9 (9.8)

Continuous variables are expressed as mean ± standard deviation or median and interquartile range according to variable distribution. *CTEPH* chronic thromboembolic pulmonary hypertension, *BPA* balloon pulmonary angioplasty, *RAP* right atrium pressure, *PAP* pulmonary artery pressure, *PVR* pulmonary vascular resistance, *PASO*_*2*_ pulmonary arterial oxygen saturation, *MVSO*_*2*_ mixed venous oxygen saturation, *NT-proBNP* N-terminal pro-B-type natriuretic peptide, *WHO* World Health Organization

### Operation details, treated lesions and safety of bilateral BPA as compared with unilateral BPA

In a total of 210 BPA sessions, 86 BPA sessions treated arteries of both lungs, 124 unilateral BPA sessions treated arteries of only left lung or right lung (Table 2). As compared with unilateral BPA, bilateral BPA not only treated arteries from more lobes in one single session [3 (2,4) vs. 2 (1,3) lobes, p < 0.001], but also treated more arteries and lesions [8 (6,10) vs. 6 (4,8) vessels, p = 0.003; 9 (7,12) vs. 8 (5,10) lesions, p = 0.01] in a comparable operation duration [242.5 ± 69.0 vs. 239.0 ± 56.7 min, p = 0.70]. There were no significant differences in the largest balloon size used, the amount of contrast media given or the DAP between bilateral BPA and unilateral BPA [contrast media given/session, 240 (200, 295) vs. 250 (200, 300) ml, p = 0.82; DAP/session, 1613.8 (947.1, 3414.8) vs. 1708.4 (1059.0, 3686.3) μGy m^2^, p = 0.70]. As for differences in treated lesion types and location, bilateral BPA treated more web lesions [7 (4.5, 10) vs. 6 (4, 8) web lesions/session, p = 0.04] and more lesions from segmental arteries [2 (0, 4) vs. 1 (0, 2.8) lesions/session, p = 0.03] than unilateral BPA. A larger proportion of lower lobes (right/left) were treated in bilateral BPA.

**Table 2:** Operation details and treated lesions of all unilateral and bilateral BPA

	All BPA sessions (N = 210)	Unilateral BPA (N = 124)	Bilateral BPA (N = 86)	*p value
**Operation details**				
Operation duration (minutes)	240.4 ± 61.9	239.0 ± 56.7	242.5 ± 69.0	0.70
The amount of contrast media given (ml)	250 (200, 300)	250 (200, 300)	240 (200, 295)	0.82
Dose area product (μGy m^2^)	1645.0 (1043.1, 3658.5)	1708.4 (1059.0, 3686.3)	1613.8 (947.1, 3414.8)	0.70
Largest balloon size used/session (mm)	4 (3, 5)	4 (3, 5)	4 (3, 5)	0.78
Vessels treated/session	7 (5, 9)	6 (4, 8)	8 (5.5, 10)	0.003
Total number of lobes treated (Lobes treated/session)	502 [2 (2, 3)]	235 [2 (1, 3)]	267 [3 (2, 4)]	< 0.001
Total number of left/right lower lobes treated (%)	281 (56.0)	120 (51.1)	161 (60.3)	0.04
Lesion types (treated lesions/session)				
Total number of treated lesions	1831 [8 (6, 11)]	1008 [8 (5, 10)]	823 [9 (7, 12)]	0.01
Ring-like stenosis	167 [0 (0, 2)]	101 [0 (0, 2)]	66 [0 (0, 2)]	0.82
Web lesions	1429 [6 (4, 9)]	778 [6 (4, 8)]	651 [7 (4.5, 10)]	0.04
Subtotal occlusion	169 [1 (0, 2)]	91 [0 (0, 1)]	78 [1 (0, 2)]	0.27
Total occlusion	66 [0 (0, 0)]	38 [0 (0, 0)]	28 [0 (0, 0)]	0.86
Lesion location (treated lesions/session)				
Lobar arteries	10	7	3	
Segmental arteries	391 [1 (0, 3)]	195 [1 (0, 2.8)]	196 [2 (0, 4)]	0.03
Subsegmental arteries	1430 [6 (4, 9)]	808 [6 (4, 9)]	622 [6 (4, 10)]	0.35

Continuous variables are expressed as mean ± standard deviation or median and interquartile range according to variable distribution

*BPA* balloon pulmonary angioplasty

*Comparison between bilateral BPA and unilateral BPA

In terms of complications (Table 3), hemoptysis, pulmonary artery perforation and lung injury were noted in 6 (4.8%), 2 (1.6%) and 4 (3.2%) unilateral BPA sessions, respectively. Hemoptysis, pulmonary artery perforation and lung injury were noted in 5 (5.8%), 2 (2.3%) and 2 (2.3%) bilateral BPA sessions, respectively. No patients suffered adverse reaction to conscious sedation/local anaesthesia, allergic reaction to contrast, or pulmonary artery dissection during the procedure. No patients suffered renal dysfunction post-procedure, or required mechanical ventilation or extracorporeal membrane oxygenation after the procedure in the present study. Overall, there was no significant difference in the occurrence of complications between bilateral BPA and unilateral BPA [9 (10.5%) vs. 12 (9.7%), p = 0.83].

**Table 3:** Occurrence of complications

	All BPA sessions (N = 210)	Unilateral BPA (N = 124)	Bilateral BPA (N = 86)	*p value
Total occurrence of complications	21 (10.0%)	12 (9.7%)	9 (10.5%)	0.83
During the procedure				
Hemoptysis	11	6	5	
Pulmonary artery perforation	4	2	2	
After the procedure				
Lung injury	6	4	2	

	First BPA sessions (N = 92)	Unilateral BPA (N = 54)	Bilateral BPA (N = 38)	
Total occurrence of complications	10 (10.9%)	5 (9.3%)	5 (13.2%)	0.80
During the procedure				
Hemoptysis	5	3	2	
Pulmonary artery perforation	3	2	1	
After the procedure				
Lung injury	2	0	2	

*BPA* balloon pulmonary angioplasty

*Comparison between bilateral BPA and unilateral BPA

### Predictive variables for the occurrence of complications

Potential factors associated with the occurrence of complications were analyzed (Table 4). Among 18 variables, WHO functional class, mPAP, PVR, NT-proBNP and the number of lobes treated/session were significantly related to the occurrence of complications according to univariate analysis. However, univariate analysis didn’t reveal bilateral BPA as a predictive factor of complications. Two variables with a significant correlation of p < 0.01 in the univariate analysis were included in the following multivariate analysis. WHO functional class (OR: 3.268, 95% CI: 1.216–8.781, p = 0.019) and PVR (OR: 1.085, 95% CI: 1.004–1.172, p = 0.040) was found to be related to the occurrence of complications. The Hosmer–Lemeshow goodness of fit test shows a significance of 0.432 for this bi-variable logistic regression model.

**Table 4:** Statistical Analysis of Variables Correlated with the Occurrence of complications

	Univariate analysis			Multivariate analysis		
	Odds Ratio	95% CI	p value	Odds Ratio	95% CI	p value
Age (years)	0.998	0.958–1.040	0.928	–	–	–
Sex (female)	0.943	0.359–2.481	0.906	–	–	–
Bilateral	1.201	0.475–3.035	0.699	–	–	–
First session	1.160	0.523–2.572	0.715	–	–	–
WHO functional class (III-IV versus I-II)	3.981	1.540–10.290	0.004	3.268	1.216–8.781	0.019
Period of BPA procedure (recent versus initial period#)	0.613	0.240–1.567	0.307	–	–	–
Mean PAP (mmHg)	1.046	1.004–1.090	0.030	–	–	–
PVR (Wood Unit)	1.109	1.029–1.195	0.007	1.085	1.004–1.172	0.040
Cardiac index (L/min/m^2^)	0.543	0.291–1.014	0.055	–	–	–
Vessels treated/session	1.126	0.977–1.297	0.102	–	–	–
Lobes treated/session	1.573	1.019–2.427	0.041	–	–	–
Lesions treated/session	1.026	0.910–1.155	0.677	–	–	–
Complex lesions treated/session	0.732	0.469–1.141	0.168	–	–	–
Largest balloon size used/session (mm)	0.654	0.428–1.001	0.050	–	–	–
NT-proBNP (pg/ml)	1.000246	1.000052–1.000440	0.013	–	–	–
The amount of contrast media given (ml)	1.000	0.993–1.007	0.949	–	–	–
Dose area product (μGy m2)	0.999919	0.999736–1.000102	0.385	–	–	–
Operation duration	1.005	0.998–1.013	0.144	–	–	–

Multivariate analysis was made using variables with significant correlation of p < 0.01 in univariate analysis

*CI* confidence intervals, *PAP* pulmonary artery pressure, *PVR* pulmonary vascular resistance, *NT-proBNP* N-terminal pro-B-type natriuretic peptide, *WHO* World Health Organization

*Complex lesions were defined as subtotal occlusions and total occlusions. #Initial period was defined by the initial 105 BPA sessions (50%) performed between January 2019 and June 2020. The recent period was defined by the recent 105 BPA sessions (50%) performed between July 2020 and November 2021

### Efficacy of bilateral BPA as compared with unilateral BPA

In 131 BPA sessions with a hemodynamics follow-up, including 49 bilateral BPA sessions and 82 unilateral BPA sessions, changes of hemodynamic parameters and NT-proBNP in a single session were compared (Table 5). Bilateral BPA had a longer interval for hemodynamics follow-up than unilateral BPA [98 (65, 185) vs. 70 (32, 113) days, p = 0.01]. Both bilateral BPA and unilateral BPA decreased mPAP, PVR and NT-proBNP in a single session [mPAP, − 4.5 ± 8.6 vs. − 3.6 ± 7.3 mmHg, p = 0.52; PVR, − 1.1 (− 3.5, 0.8) vs. − 1.8 (− 5.2, 0.3) Wood units, p = 0.21; NT-proBNP, − 77.8 (− 563.8, 0.8) vs. − 66.3 (− 357.7, 4.7) pg/ml, p = 0.98], but without significant difference between the two groups. Improvement of CO and CI were not so apparent in bilateral BPA as compared with unilateral BPA in a single BPA session [CO, 0.0 (− 0.7, 0.9) vs. 0.4 (− 0.2, 1.4) L/min, p = 0.12; CI, 0.1 (− 0.4, 0.6) vs. 0.2 (− 0.2, 0.8) L/min/m^2^, p = 0.19].

**Table 5:** Changes of hemodynamics and NT-proBNP in all unilateral and bilateral BPA

	All BPA sessions (N = 131)	Unilateral BPA (N = 82)	Bilateral BPA (N = 49)	*p value
Interval between BPA (days)	80 (48, 131)	70 (32, 113)	98 (65, 185)	0.01
Changes of hemodynamics				
Heart rate	− 4.4 ± 12.1	− 4.6 ± 12.0	− 4.0 ± 12.5	0.81
Mean RAP (mmHg)	− 1.0 (− 4.0, 2.0)	− 1.0 (− 4.0, 2.0)	0.0 (− 3.0, 2.5)	0.44
Systolic PAP (mmHg)	− 6.9 ± 13.7	− 6.0 ± 12.6	− 8.5 ± 15.5	0.33
Diastolic PAP (mmHg)	− 2.4 ± 6.3	− 2.2 ± 6.8	− 2.6 ± 5.6	0.75
Mean PAP (mmHg)	− 3.9 ± 7.8	− 3.6 ± 7.3	− 4.5 ± 8.6	0.52
Cardiac output (L/min)	0.2 (− 0.4, 1.2)	0.4 (− 0.2, 1.4)	0.0 (− 0.7, 0.9)	0.12
Cardiac index (L/min/m^2^)	0.1 (− 0.3, 0.7)	0.2 (− 0.2, 0.8)	0.1 (− 0.4, 0.6)	0.19
PVR (Wood Unit)	− 1.8 (− 4.2, 0.5)	− 1.8 (− 5.2, 0.3)	− 1.1 (− 3.5, 0.8)	0.21
PASO_2_ (%)	1.9 ± 7.3	2.6 ± 7.1	0.7 ± 7.5	0.16
MVSO_2_ (%)	0.5 ± 4.2	0.5 ± 4.0	0.5 ± 4.6	0.99
Changes of NT-proBNP (pg/ml)	− 75.0 (− 486.7, 1.7)	− 66.3 (− 357.7, 4.7)	− 77.8 (− 563.8, 0.8)	0.98

Continuous variables are expressed as mean ± standard deviation or median and interquartile range according to variable distribution

*BPA* balloon pulmonary angioplasty, *RAP* right atrium pressure, *PAP* pulmonary artery pressure, *PVR* pulmonary vascular resistance, *PASO*_*2*_ pulmonary arterial oxygen saturation, *MVSO*_*2*_ mixed venous oxygen saturation, *NT-proBNP* N-terminal pro-B-type natriuretic peptide, *WHO* World Health Organization

*Comparison between bilateral BPA and unilateral BPA

### Operation details, treated lesions and safety of bilateral BPA as compared with unilateral BPA in the initial BPA session

We also evaluate the operation details, treated lesions and safety of bilateral BPA in the initial BPA session, since interventional radiologists tend to take more conservative tactics in patients’ initial BPA (Fig. 1B). Patients’ characteristics before the initial BPA are also comparable between patients who underwent bilateral BPA in the initial session and those who underwent unilateral BPA (Table 6). 92 initial BPA sessions, including 54 unilateral BPA session and 38 bilateral BPA, were included for evaluation. Bilateral BPA treated arteries from more lobes in the initial session as compared with unilateral BPA [3 (2, 4) vs. 2 (1, 2) lobes, p < 0.001]. Meanwhile, bilateral BPA treated more arteries and lesions [8.0 (5.8, 9.3) vs. 6.0 (4.0, 8.0) vessels, p = 0.04; 9 (6, 12) vs. 7 (4, 10) lesions, p = 0.02] in the initial session as compared with unilateral BPA in a comparable operation duration [229.0 ± 70.8 vs. 232.5 ± 58.4 min, p = 0.80]. There were no significant differences in the largest balloon size used, the amount of contrast media given or the DAP between bilateral BPA and unilateral BPA [contrast media given/session, 230.0 (200.0, 260.0) vs. 230.0 (180.0, 300.0) ml, p = 1.00; DAP/session, 1560.1 (916.7, 3480.6) vs. 1550.6 (1090.0, 3485.4) μGy m^2^, p = 0.90]. As for differences in treated lesion types and location, bilateral BPA also treated more web lesions [7.5 (5, 10) vs. 5 (3, 7) web lesions/session, p = 0.004] in the initial session than unilateral BPA. There is no significant difference in treated lesions distribution between bilateral BPA and unilateral BPA in the initial session.

**Table 6:** Operation details and treated lesions of the initial BPA session

	First BPA sessions (N = 92)	Unilateral BPA (N = 54)	Bilateral BPA (N = 38)	*p value
Operation details				
Operation duration (minutes)	231.1 ± 63.5	232.5 ± 58.4	229.0 ± 70.8	0.80
The amount of contrast media given (ml)	220.0 (180.0, 270.0)	230.0 (180.0, 300.0)	230.0 (200.0, 260.0)	1.00
Dose area product (μGy m^2^)	1550.6 (951.1, 3450.1)	1550.6 (1090.0, 3485.4)	1560.1 (916.7, 3480.6)	0.90
Largest balloon size used/session (mm)	3.5 (3.0, 4.5)	4.0 (2.9, 4.6)	3.0 (3.0, 4.3)	0.62
Vessels treated/session	7.0 (4.3, 9.0)	6.0 (4.0, 8.0)	8.0 (5.8, 9.3)	0.04
Total number of lobes treated (Lobes treated/session)	220 [2 (2, 3)]	103 [2 (1, 2)]	117 [3 (2, 4)]	< 0.001
Total number of left/right lower lobes treated (%)	124 (56.3)	51 (49.5)	73 (62.4)	0.06
Lesion type (treated lesions/session)				
Total number of treated lesions	738 [8 (5, 10)]	393 [7 (4, 10)]	345 [9 (6, 12)]	0.02
Ring-like stenosis	64 [0 (0, 1)]	46 [0 (0, 2)]	18 [0 (0, 1)]	0.07
Web lesions	575 [6 (3.3, 8)]	289 [5 (3, 7)]	286 [7.5 (5, 10)]	0.004
Subtotal occlusion	71 [1 (0, 1)]	41 [1 (0, 1)]	30 [0.5 (0, 2)]	0.99
Total occlusion	28 [0 (0, 0)]	17 [0 (0, 0)]	11 [0 (0, 0)]	0.72
Lesion location (treated lesions/session)				
Lobar arteries	5	4	1	
Segmental arteries	180 [2 (0, 3)]	94 [1.5 (0, 3)]	86 [2 (0, 4)]	0.34
Subsegmental arteries	553 [5 (4, 8)]	295 [5 (3, 8)]	258 [6 (4, 10)]	0.13

Continuous variables are expressed as mean ± standard deviation or median and interquartile range according to variable distribution

*BPA* balloon pulmonary angioplasty

*Comparison between bilateral BPA and unilateral BPA

In terms of complications, hemoptysis and pulmonary artery perforation were noted in 3 (5.6%) and 2 (3.7%) unilateral BPA sessions, respectively. Hemoptysis, pulmonary artery perforation and lung injury were noted in 2 (5.3%), 1 (2.6%) and 2 (5.3%) bilateral BPA sessions, respectively. Overall, no significant difference was shown in the occurrence of complications between bilateral BPA and unilateral BPA [5 (13.2%) vs. 5 (9.3%), p = 0.80].

### Efficacy of bilateral BPA as compared with unilateral BPA in the initial BPA session

In 62 initial BPA sessions for CTEPH patients with a hemodynamic follow-up, changes of hemodynamic parameters and NT-proBNP were compared between 23 bilateral BPA sessions and 39 unilateral BPA sessions (Table 7). Bilateral BPA had a longer interval for hemodynamics follow-up than unilateral BPA [95 (63, 185) vs. 64 (30, 94) days, p = 0.02]. Overall, both bilateral BPA and unilateral BPA improved hemodynamics in the initial BPA session. However, differences in the improvement of hemodynamic didn’t show statistical significance between the two groups [mPAP, − 6.8 ± 9.3 vs. − 4.1 ± 7.4 mmHg, p = 0.22; PVR, − 1.5 (− 5.8, 0.9) vs. − 1.8 (− 4.2, 0.7) Wood units, p = 0.99].

**Table 7:** Clinical baseline characteristics, Changes of hemodynamics and NT-proBNP in the initial BPA session

	First BPA sessions	Unilateral BPA	Bilateral BPA	*p value
Clinical baseline characteristics (N)	92	54	38	
Age (years)	59 ± 12	60 ± 13	56 ± 11	0.10
Female (%)	63 (68%)	40 (74%)	23 (61%)	0.17
Heart rate	80.5 ± 13.2	79.0 ± 13.3	82.7 ± 12.8	0.20
Mean PAP (mmHg)	43.0 ± 13.6	41.9 ± 13.8	44.5 ± 13.3	0.37
PVR (Wood Unit)	8.9 (4.9, 15.1)	8.3 (5.1, 15.9)	9.9 (4.6, 14.1)	0.92
Cardiac output (L/min)	3.6 (2.7, 5.0)	3.4 (2.6, 5.5)	3.8 (3.3, 5.0)	0.18
Cardiac index (L/min/m^2^)	2.2 (1.7, 3.2)	2.1 (1.7, 3.2)	2.5 (2.1, 3.2)	0.22
NT-proBNP (pg/ml)	541.1 (120.0, 1823.8)	321.1 (120.0, 1816.0)	950.3 (110.4, 2092.3)	0.34
WHO functional class (I–II/III–IV)	56.5%/43.5%	57.4%/42.6%	55.3%/44.7%	0.84
Interval between BPA (days)	67 (35, 107)	64 (30, 94)	95 (63, 185)	0.02
Changes of hemodynamics (N)	62	39	23	
Heart rate	− 5.3 ± 11.8	− 3.7 ± 10.9	− 7.9 ± 12.9	0.18
Mean RAP (mmHg)	0.0 (− 4.0, 3.0)	0.0 (− 4.0, 3.3)	− 1.0 (− 3.5, 2.0)	0.54
Systolic PAP (mmHg)	− 8.9 ± 14.7	− 7.3 ± 13.3	− 11.6 ± 16.8	0.28
Diastolic PAP (mmHg)	− 2.9 ± 6.9	− 2.4 ± 7.4	− 3.8 ± 5.9	0.45
Mean PAP (mmHg)	− 5.1 ± 8.2	− 4.1 ± 7.4	− 6.8 ± 9.3	0.22
Cardiac output (L/min)	0.0 (− 0.6, 0.9)	0.0 (− 0.8, 0.9)	0.0 (− 0.5, 0.9)	0.62
Cardiac index (L/min/m^2^)	0.0 (− 0.4, 0.5)	0.0 (− 0.5, 0.5)	0.1 (− 0.2, 0.5)	0.41
PVR (Wood Unit)	− 1.8 (− 4.5, 0.8)	− 1.8 (− 4.2, 0.7)	− 1.5 (− 5.8, 0.9)	0.99
PASO_2_ (%)	1.1 ± 7.5	0.9 ± 6.6	1.5 ± 8.9	0.77
MVSO_2_ (%)	0.1 ± 4.4	0.4 ± 4.2	− 0.2 ± 4.9	0.62
Changes of NT-proBNP (pg/ml)	− 131.3 (− 937.8, 2.5)	− 109.3 (− 725.4, − 2.4)	− 488.0 (− 1359.2, 19.5)	0.53

Continuous variables are expressed as mean ± standard deviation or median and interquartile range according to variable distribution

*BPA* balloon pulmonary angioplasty, *RAP* right atrium pressure, *PAP* pulmonary artery pressure, *PVR* pulmonary vascular resistance, *PASO*_*2*_ pulmonary arterial oxygen saturation, *MVSO*_*2*_ mixed venous oxygen saturation, *NT-proBNP* N-terminal pro-B-type natriuretic peptide, *WHO* World Health Organization

*Comparison between bilateral BPA and unilateral BPA

### Overall effect of BPA treatment

Table 8 shows the overall effect of BPA on exercise capacity and hemodynamics. In 24 patients with CTEPH [18 (75%) female, mean age 59 ± 10 year-old] who complete all BPA procedures with a median follow-up of 115 (81, 177) days, a significant improvement in hemodynamic parameters including mPAP, PVR, CO, CI were observed [mPAP, 40.6 ± 13.8–28.5 ± 8.0 mmHg, p = 0.001; PVR, 8.0 (3.7,17.0) to 4.0 (2.9, 5.4) Wood units, P = 0.007; CO, 3.5 (2.6, 5.4) to 4.7 (3.7, 5.8) L/min, p = 0.017; CI, 2.2 (1.8, 3.4) to 2.9 (2.5, 3.5) L/min/m^2^, p = 0.044]. NT-proBNP and exercise capacity were also improved in follow-up (Table 8).

**Table 8:** Clinical and hemodynamic effect of BPA treatment in 24 CTEPH patients

	24 CTEPH patients		p
	Before BPA	After BPA	
Patient characteristics			
Age (years)	59 ± 10		–
Female (%)	18 (75%)		–
Number of BPA sessions/patient	3 (2,4)		–
Duration to follow-up (days)	115 (81, 177)		–
PAH targeted therapy (%)	11 (45.8%)		
Hemodynamics			
Heart rate	79.8 ± 15.3	69.5 ± 10.1	0.009
Mean RAP (mmHg)	7.9 ± 4.0	5.5 ± 3.4	0.062
Systolic PAP (mmHg)	70.8 ± 25.0	50.3 ± 14.2	0.001
Diastolic PAP (mmHg)	23.5 ± 9.1	15.8 ± 6.0	0.001
Mean PAP (mmHg)	40.6 ± 13.8	28.5 ± 8.0	0.001
PVR (Wood unit)	8.0 (3.7,17.0)	4.0 (2.9, 5.4)	0.007
Cardiac output (L/min)	3.5 (2.6, 5.4)	4.7 (3.7, 5.8)	0.017
Cardiac index (L/min/m^2^)	2.2 (1.8, 3.4)	2.9 (2.5, 3.5)	0.044
PASO_2_ (%)	64.1 ± 9.5	68.7 ± 7.2	0.068
MVSO_2_ (%)	93.7 ± 3.4	95.2 ± 3.0	0.116
NT-proBNP (pg/ml)	171.0 (71.5, 1569.0)	50.6 (26.5, 113.4)	0.002
Exercise capacity			
WHO functional class (I–II/III–IV, %)	50.0%/50.0%	95.8%/4.2%	< 0.001
Absolute change of 6MWD (meters)	88.0 ± 55.5		< 0.001

Continuous variables are expressed as mean ± standard deviation or median and interquartile range according to variable distribution

*CTEPH* chronic thromboembolic pulmonary hypertension, *BPA* balloon pulmonary angioplasty, *RAP* right atrium pressure, *PAP* pulmonary artery pressure, *PVR* pulmonary vascular resistance, *PASO*_*2*_ pulmonary arterial oxygen saturation, *MVSO*_*2*_ mixed venous oxygen saturation, *NT-proBNP* N-terminal pro-B-type natriuretic peptide, *WHO* World Health Organization, *6MWD* 6-min walk distance

## Discussion

In the present study, we have demonstrated in 210 BPA sessions that: (1) Bilateral BPA, while treating more lobes, arteries and lesions from both lungs in one session, shares a similar occurrence of complications with unilateral BPA; (2) the operation duration, the amount of contrast media given and the DAP per session didn’t differ significantly between bilateral BPA and unilateral BPA, implying that bilateral BPA might not increase the radiation burden on patients; (3) bilateral BPA, although treating more lobes, arteries and lesions in one session, with a longer duration to follow-up, didn’t improve hemodynamics or NT-proBNP better than unilateral BPA; (4) the number of lobes treated/session, but not bilateral BPA, was revealed as predictive factors of complications.

During the past decade, the efficacy of BPA treatment on hemodynamics and exercise capacity has been proved in a series of studies worldwide [15]. Our results of 24 CTEPH patients who finished all BPA procedures with a hemodynamic follow-up also show similar hemodynamics effect to the results achieved in European centers [16, 17], but slightly worse than that achieved in Japan [18, 19], possibly due to a higher level of baseline mPAP and PVR in our cohort. Meanwhile, consideration has been focusing on the refinement of BPA procedure in the aim of lowering radiation exposure, curbing the risk of perioperative complications while avoiding post-procedural mortality [8]. Following the pioneer study of BPA for CTEPH patients by Feinstein et al., which report a high occurrence of BPA-related reperfusion pulmonary oedema (RPO) [20], several Japanese centers have reported the overall efficacy of BPA treatments since 2012, with relatively low rates of complications benefiting from refined BPA procedure performed with smaller sized balloons for fewer lobes per procedure [9, 21, 22]. Ever since, some centers have adopted a more conservative manner, by treating arteries of one single lobe in the initial BPA session. In 2014, Fukui et al. reported zero death and no major complications in 20 CTEPH patients treated with BPA (3.2 ± 0.9 sessions/patient) by treating one single lobe in the initial BPA session [10]. On the other hand, in 2018, Velázquez et al. reported one death and a complication rate of 28.2% in 156 BPA sessions for 46 CTEPH patients even when only one lobe was treated in one single BPA session until mPAP ≤ 35 mmHg [23], implying that the occurrence of perioperative complication might be dictated by various factors other than the number of vessels or lobes treated per session. Although a study from Japan has described the strategy of treating both lungs in one BPA session when mPAP < 35 mmHg in 2012 [22], the safety of bilateral BPA has never been fully investigated. In the present study, we classified BPA complication according to the 6th World Symposium on Pulmonary Hypertension (2018) and compared bilateral BPA with unilateral BPA performed in our center. We have found that the occurrence of complications is similar between the two group, while the overall rate of complications was comparable with a large cohort from France (10%, 21/210 events vs. 11.2%, 113/1006 events) [16]. What is more, logistic regression analysis didn’t reveal bilateral BPA as a factor related to the occurrence of perioperative complications.

RPO is a common complication of BPA, and previous study has found from multivariate regression analysis that procedures performed in cases of first session, or in patients with greater clinical severity at baseline had a higher risk of RPO [24]. While previous study and practice have been cautious about the number of lesions, vessels or lobes treated per session and the total changes of pulmonary blood flow per session [7, 8, 24], whether increasing the number of lesions, vessels or lobes treated per session is related to a higher occurrence of complication have never been discovered. In this study, apart from mPAP, PVR and NT-proBNP, we have for the first time identified WHO functional class and the number of lobes treated/session as factors related to the occurrence of perioperative complications. Interestingly, in this study, patients’ baseline hemodynamics before the first session are comparable between bilateral BPA and unilateral BPA. Bilateral BPA, performed in the first session in patients with high levels of mPAP and PVR, didn’t significantly increase the occurrence of complications including RPO and hemoptysis. Besides, in a comparable operation duration and a same limited amount of contrast media given per session, bilateral BPA treated more lobes, arteries and lesions in one session. Although bilateral BPA was not identified as a predictor of perioperative complications in the present study, it should be cautious that the practice of bilateral BPA has made it inevidable to treat at least two lobes in one single session, which might be related to a higher occurrence of complications.

In practice, we didn’t limit the number of lobes treated per session, but focusing more on selecting appropriate lesions for dilation on the other hand. Ring-like stenosis and web lesions from all lobes (preferably right lower lobe at first) were targeted in priority, which were more easily treated with lower rates of complication [14]. In addition, targeting such lesions is more time-saving than targeting a total occlusion lesion, which partly explain why more arteries were treated in bilateral BPA, since more complicated lesions were often focused in a unilateral BPA under such strategy. By targeting simpler lesions at first, we are able to improve patients’ hemodynamics and cardiac function, offering a better patient condition for their next BPA, for more complex lesions. In the end, all accessible lesions would be treated, with an overall improvement of hemodynamics and exercise capacity.

A Japanese perspective has recommended that radiation exposure, fluoroscopy time, and the amount of contrast media in each session should not exceed 1000 mGy, 60 min, and 300 ml, respectively [8]. In practice, we didn’t limit the time of procedure, because BPA is always accompanied by two RHC before and after each procedure, and sometimes with the application of optical coherence tomography in some sessions. In the early stage of practicing BPA in our center (the learning curve period), contrast media given in some BPA sessions exceeded 300 ml, which was also shown in our previously published study [12]. After the learning curve period, with the improvement of BPA procedure and the application of 3D images derived from DynaCT angiographic reconstruction for the selection of target lesions [12], contrast media given per session was decreased significantly (< 300 ml), and the amount of contrast media given in most procedures performed more recently was 200–250 ml. A learning curve period for this complex procedure, to balance the optimal treatment effect and the radiation burden for patients and the radiologists, is unavoidable.

Several limitations should be mentioned in this study. First of all, we performed a single-center retrospective study with a relatively small number of patients and BPA sessions. Therefore, the findings in this study need to be further validated in future multi-center studies with a larger cohort. Secondly, we compared the changes of hemodynamics and NT-proBNP between two consecutive BPA sessions as the efficacy of each BPA session, with an interval of 1–12 month. However, hemodynamics effect takes time to develop after BPA, and the additional hemodynamics effect of the first BPA session might surface in later sessions. Patients included in this study are for BPA treatment rather than for a scientific purpose, so hemodynamics effect in each BPA sessions shows a real-world condition in our study. Thirdly, chest CT was not routinely performed in all patients post-BPA, which might underestimate the rate of lung injury (RPO) in all BPA sessions. In our center, only when patients report adverse events post-BPA (hemoptysis, severe cough, hypoxemia, or tachycardia), or when lung opacities were found on chest X-ray, that a chest CT would be performed subsequently. Lastly, although bilateral BPA treated more arteries from more lobes in a single session, we are unable to demonstrate whether bilateral BPA reduces the number of BPA procedures needed per patient, since most patients in our study received both bilateral BPA and unilateral BPA according to lesions type and location in their whole BPA treatment. It would be meaningful if such hypothesis, that bilateral BPA reduces the number of procedures needed per patient and thus reducing the financial costs in patients, be demonstrated and confirmed in further study.

## Conclusions

Our study provides the first comprehensive evaluation of the safety and efficacy of bilateral BPA as compared with unilateral BPA. Bilateral BPA, while treating more lobes, arteries and lesions than unilateral BPA in one single session, might not increase the operation duration, the occurrence of perioperative complications or radiation burden of procedures. Our findings bring new insights into the standardization of BPA procedures by focusing more on the lesions but not only the number of lobes, arteries and lesions treated per session. Further multi-center studies with a large number of patients are needed to evaluated the potential benefits or risks of bilateral BPA.

## Data Availability

Data sharing is not applicable to this article as no datasets were generated or analyzed during the current study.

## Abbreviations

BPA: Balloon pulmonary angioplasty
CTEPH: Chronic thromboembolic pulmonary hypertension
CI: Cardiac index
CO: Cardiac output
DAP: Dose area product
mPAP: Mean pulmonary artery pressure
MVSO2: Mixed venous oxygen saturation
NT-proBNP: N-terminal pro-B-type natriuretic peptide
PAH: Pulmonary arterial hypertension
PAP: Pulmonary artery pressure
PASO2: Pulmonary arterial oxygen saturation
PEA: Pulmonary endarterectomy
PVR: Pulmonary vascular resistance
RAP: Right atrial pressure
RHC: Right heart catheterization
RPO: Reperfusion pulmonary oedema
WHO: World Health Organization
6MWD: 6-Minute walk distance

## References

[1] Kim NH, Delcroix M, Jais X, Madani MM, Matsubara H, Mayer E, Ogo T, Tapson VF, Ghofrani HA, Jenkins DP (2019). Chronic thromboembolic pulmonary hypertension. Eur Respir J.

[2] Simonneau G, Torbicki A, Dorfmüller P, Kim N (2017). The pathophysiology of chronic thromboembolic pulmonary hypertension. Eur Respir Rev.

[3] Galiè N, Humbert M, Vachiery JL, Gibbs S, Lang I, Torbicki A, Simonneau G, Peacock A, Vonk Noordegraaf A, Beghetti M, Ghofrani A, Gomez Sanchez MA, Hansmann G, Klepetko W, Lancellotti P, Matucci M, McDonagh T, Pierard LA, Trindade PT, Zompatori M, Hoeper M, ESC Scientific Document Group (2016). 2015 ESC/ERS Guidelines for the diagnosis and treatment of pulmonary hypertension: the Joint Task Force for the Diagnosis and Treatment of Pulmonary Hypertension of the European Society of Cardiology (ESC) and the European Respiratory Society (ERS): endorsed by: Association for European Paediatric and Congenital Cardiology (AEPC), International Society for Heart and Lung Transplantation (ISHLT). Eur Heart J.

[4] Pepke-Zaba J, Delcroix M, Lang I, Mayer E, Jansa P, Ambroz D, Treacy C, D'Armini AM, Morsolini M, Snijder R, Bresser P, Torbicki A, Kristensen B, Lewczuk J, Simkova I, Barberà JA, de Perrot M, Hoeper MM, Gaine S, Speich R, Gomez-Sanchez MA, Kovacs G, Hamid AM, Jaïs X, Simonneau G (2011). Chronic thromboembolic pulmonary hypertension (CTEPH): results from an international prospective registry. Circulation.

[5] Ghofrani HA, D'Armini AM, Grimminger F, Hoeper MM, Jansa P, Kim NH, Mayer E, Simonneau G, Wilkins MR, Fritsch A, Neuser D, Weimann G, Wang C, CHEST-1 Study Group (2013). Riociguat for the treatment of chronic thromboembolic pulmonary hypertension. N Engl J Med.

[6] Ghofrani HA, Simonneau G, D'Armini AM, Fedullo P, Howard LS, Jaïs X, Jenkins DP, Jing ZC, Madani MM, Martin N, Mayer E, Papadakis K, Richard D, Kim NH, MERIT study investigators (2017). Macitentan for the treatment of inoperable chronic thromboembolic pulmonary hypertension (MERIT-1): results from the multicentre, phase 2, randomised, double-blind, placebo-controlled study. Lancet Respir Med.

[7] Lang I, Meyer BC, Ogo T, Matsubara H, Kurzyna M, Ghofrani HA, Mayer E, Brenot P (2017). Balloon pulmonary angioplasty in chronic thromboembolic pulmonary hypertension. Eur Respir Rev.

[8] Kataoka M, Inami T, Kawakami T, Fukuda K, Satoh T (2019). Balloon pulmonary angioplasty (percutaneous transluminal pulmonary angioplasty) for chronic thromboembolic pulmonary hypertension: a japanese perspective. JACC Cardiovasc Interv.

[9] Kataoka M, Inami T, Hayashida K, Shimura N, Ishiguro H, Abe T, Tamura Y, Ando M, Fukuda K, Yoshino H, Satoh T (2012). Percutaneous transluminal pulmonary angioplasty for the treatment of chronic thromboembolic pulmonary hypertension. Circ Cardiovasc Interv.

[10] Fukui S, Ogo T, Morita Y, Tsuji A, Tateishi E, Ozaki K, Sanda Y, Fukuda T, Yasuda S, Ogawa H, Nakanishi N (2014). Right ventricular reverse remodelling after balloon pulmonary angioplasty. Eur Respir J.

[11] Jin Q, Luo Q, Yang T, Zeng Q, Yu X, Yan L, Zhang Y, Zhao Q, Ma X, An C, Xiong C, Zhao Z, Liu Z (2019). Improved hemodynamics and cardiopulmonary function in patients with inoperable chronic thromboembolic pulmonary hypertension after balloon pulmonary angioplasty. Respir Res.

[12] Lin JL, Chen HM, Lin FC, Li JY, Xie CX, Guo WL, Huang XF, Hong C (2020). Application of DynaCT angiographic reconstruction in balloon pulmonary angioplasty. Eur Radiol.

[13] Hong C, Luo FQ, Liu CL, Zhong NS, Li JY, Wang W (2018). Clinical study of optical coherence tomography in the diagnosis of peripheral pulmonary artery thrombus. Thromb Res.

[14] Kawakami T, Ogawa A, Miyaji K, Mizoguchi H, Shimokawahara H, Naito T, Oka T, Yunoki K, Munemasa M, Matsubara H (2016). Novel angiographic classification of each vascular lesion in chronic thromboembolic pulmonary hypertension based on selective angiogram and results of balloon pulmonary angioplasty. Circ Cardiovasc Interv.

[15] Zhang L, Bai Y, Yan P, He T, Liu B, Wu S, Qian Z, Li C, Cao Y, Zhang M (2021). Balloon pulmonary angioplasty vs. pulmonary endarterectomy in patients with chronic thromboembolic pulmonary hypertension: a systematic review and meta-analysis. Heart Fail Rev.

[16] Brenot P, Jaïs X, Taniguchi Y, Garcia Alonso C, Gerardin B, Mussot S, Mercier O, Fabre D, Parent F, Jevnikar M, Montani D, Savale L, Sitbon O, Fadel E, Humbert M, Simonneau G (2019). French experience of balloon pulmonary angioplasty for chronic thromboembolic pulmonary hypertension. Eur Respir J.

[17] Olsson KM, Wiedenroth CB, Kamp JC, Breithecker A, Fuge J, Krombach GA, Haas M, Hamm C, Kramm T, Guth S, Ghofrani HA, Hinrichs JB, Cebotari S, Meyer K, Hoeper MM, Mayer E, Liebetrau C, Meyer BC (2017). Balloon pulmonary angioplasty for inoperable patients with chronic thromboembolic pulmonary hypertension: the initial German experience. Eur Respir J.

[18] Aoki T, Sugimura K, Tatebe S, Miura M, Yamamoto S, Yaoita N, Suzuki H, Sato H, Kozu K, Konno R, Miyata S, Nochioka K, Satoh K, Shimokawa H (2017). Comprehensive evaluation of the effectiveness and safety of balloon pulmonary angioplasty for inoperable chronic thrombo-embolic pulmonary hypertension: long-term effects and procedure-related complications. Eur Heart J.

[19] Ogawa A, Satoh T, Fukuda T, Sugimura K, Fukumoto Y, Emoto N, Yamada N, Yao A, Ando M, Ogino H, Tanabe N, Tsujino I, Hanaoka M, Minatoya K, Ito H, Matsubara H (2017). Balloon pulmonary angioplasty for chronic thromboembolic pulmonary hypertension: results of a multicenter registry. Circ Cardiovasc Qual Outcomes.

[20] Feinstein JA, Goldhaber SZ, Lock JE, Ferndandes SM, Landzberg MJ (2001). Balloon pulmonary angioplasty for treatment of chronic thromboembolic pulmonary hypertension. Circulation.

[21] Sugimura K, Fukumoto Y, Satoh K, Nochioka K, Miura Y, Aoki T, Tatebe S, Miyamichi-Yamamoto S, Shimokawa H (2012). Percutaneous transluminal pulmonary angioplasty markedly improves pulmonary hemodynamics and long-term prognosis in patients with chronic thromboembolic pulmonary hypertension. Circ J.

[22] Mizoguchi H, Ogawa A, Munemasa M, Mikouchi H, Ito H, Matsubara H (2012). Refined balloon pulmonary angioplasty for inoperable patients with chronic thromboembolic pulmonary hypertension. Circ Cardiovasc Interv.

[23] Velázquez M, Albarrán A, Hernández I, López-Gude MJ, Sarnago F, Martín R, Arribas F, Escribano P (2019). Balloon pulmonary angioplasty for inoperable patients with chronic thromboembolic pulmonary hypertension. Observational study in a referral unit. Rev Esp Cardiol (Engl Ed)..

[24] Inami T, Kataoka M, Shimura N, Ishiguro H, Yanagisawa R, Taguchi H, Fukuda K, Yoshino H, Satoh T (2013). Pulmonary edema predictive scoring index (PEPSI), a new index to predict risk of reperfusion pulmonary edema and improvement of hemodynamics in percutaneous transluminal pulmonary angioplasty. JACC Cardiovasc Interv.

